# Valorization of Hot Trub: Extraction and Nanoparticle Encapsulation for Antitumor Applications

**DOI:** 10.1002/cbdv.71827

**Published:** 2026-10-12

**Authors:** Lilian Tatiani Dusman Tonin, Isabella Salvat Luccas, Gabriela Barbosa da Silva, Giovanna Martins Domingos, Grazielle Jesus dos Santos, Elisângela Düsman, Ana Beatriz Sousa Santana, Patricia Aline Bressiani, Ágatha Giovanna Fragoso, Angélica Priscila Parussolo Tonin, Eduardo César Meurer

**Affiliations:** ^1^ Postgraduate Program in Chemical Engineering Federal Technological University of Paraná (UTFPR) Apucarana Paraná Brazil; ^2^ Chemical Engineering Course Coordination Federal Technological University of Paraná (UTFPR) Apucarana Paraná Brazil; ^3^ Department of Chemistry and Biology Federal Technological University of Paraná (UTFPR) Francisco Beltrão Paraná Brazil; ^4^ Laboratory Fenn of Mass Spectrometry Department of Chemistry Federal University of Paraná (UFPR) Jandaia do Sul Paraná Brazil

**Keywords:** cancer, encapsulation, optimization, phenolic compounds, sun protection factor, xanthohumol

## Abstract

The brewing industry generates large amounts of waste containing bioactive compounds with potential applications in the pharmaceutical and cosmetic industries. This study optimized the extraction of bioactive compounds from hot trub using 70:30 (v/v) ethanol:water as the extraction solvent, with time and temperature optimized through an experimental design. The optimized extract exhibited high antioxidant activity and was rich in phenolic acids, flavonoids, and xanthohumol. The extract demonstrated promising photoprotective properties, with a sun protection factor of 13.84, suggesting its potential for dermocosmetic use. The extract was encapsulated in zein nanoparticles, including chitosan‐coated and sodium caseinate‐coated variants, to enhance stability, bioavailability, and bioactivity. The effect of the encapsulated extract on cell viability was evaluated against the MCF‐7 breast cancer cell line. Results indicated that encapsulation in zein nanoparticles improved the in vitro effect of the extract on MCF‐7 cell viability, with chitosan and sodium caseinate coatings enhancing this effect. These findings highlight hot trub as a sustainable source of bioactive compounds with potential for dermocosmetic applications and as a basis for further investigation in cancer research. Future studies should focus on evaluating the long‐term stability of the nanoparticles and on assessing their biological activity in more complex models.

## Graphical abstract

Hot trub, a brewing by‐product, is valorized through optimized extraction of phenolic compounds and encapsulation in zein nanoparticles. The polyphenol‐rich extract shows antioxidant and photoprotective activity (SPF 13.84), while zein‐based nanocarriers, particularly chitosan‐ and caseinate‐coated variants, extend its effect on MCF‐7 breast cancer cell viability, positioning this waste as a sustainable source of bioactive agents.

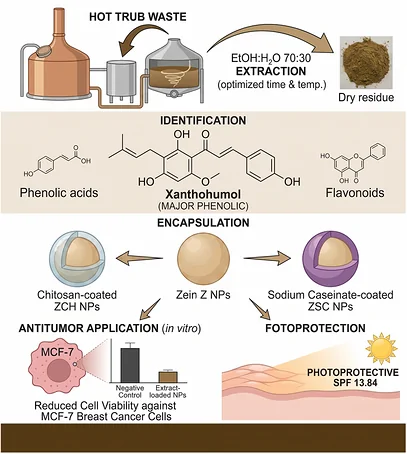

## 1 Introduction

Free radicals are major contributors to cellular damage and oxidative stress, which are strongly associated with aging, various cancers, and neurodegenerative disorders [1]. To counteract these effects, cells have developed an antioxidant protection system involving a variety of endogenous and exogenous compounds. Natural products and their by‐products provide important exogenous antioxidants, especially phenolic compounds, which constitute a major group of phytonutrients [2].

Polyphenols exhibit a broad spectrum of biological properties, including antioxidant, anti‐inflammatory, and anticancer activities. Studies indicate that dietary intake of polyphenol‐rich foods may help prevent cancer, cardiovascular diseases, and neurodegenerative disorders. Because of these properties, polyphenols are being explored for use in preventive and therapeutic medicine, functional foods and beverages, natural preservatives, and dermocosmetic products [2, 3]. In this context, plant extracts rich in phenolic compounds have also been successfully employed as reducing and capping agents in the green synthesis of bioactive nanoparticles with enhanced antioxidant and antimicrobial properties [4].

Agro‐industrial by‐products, particularly those generated by the brewing industry, are abundant and still underexploited sources of phenolic compounds. The Brazilian beer market produced 15.4 billion liters of beer in 2023, making Brazil the third‐largest producer, behind China and the United States [5]. The brewing industry generates large amounts of solid and liquid waste, raising significant ecological and economic management concerns. Major by‐products include brewer's spent grain, spent hops, and spent yeast, in addition to hot trub, all of which are rich in bioactive compounds with potential applications in the food, pharmaceutical, and cosmetic industries [6]. Hot trub is separated from the wort during the whirlpool stage prior to fermentation and consists mainly of hop polyphenols and precipitated wort proteins. Approximately 85% of the hops added to beer end up in by‐products, and the production of 100 L of beer yields about 300 g of hot trub [7, 8]. Hop (*Humulus lupulus* L.) is added during wort boiling and imparts bitterness (via humulones and lupulones), aroma, flavor, and contributes to its stability [9].

Bocquet et al. [10] reported that hops contain over 300 phytochemicals, including terpenes, chalcones, flavonoids, tannins, lignans, phenolic acid derivatives, phloroglucinol derivatives, and nitrogen‐containing compounds. Many hop phenolic compounds precipitate with proteins during wort boiling, enriching the hot trub with bioactive molecules [8]. Costa et al. [11] identified humulones, lupulones, isoxanthohumol, and xanthohumol in ultrasound‐assisted ethanol extracts of hot trub, and demonstrated high antioxidant activity of the crude ethanol extract and antibacterial activity of the hexane fraction against Gram‐positive bacteria, with relatively low cytotoxicity.

Tatullo et al. [12] isolated a bioactive polyphenolic fraction from brewing wastewater, consisting mainly of hot trub, and demonstrated its significant antioxidant and antitumor activities, including improved mitochondrial function and reduced oxidative stress in tumor cells, effects attributed to its high content of phenolic compounds, particularly flavonoids.

In addition to its antioxidant and antimicrobial potential, hot trub has been investigated as a source of essential oils for insect repellents [13]. Acid‐assisted extraction also recovers pectins from hot trub for use as gelling and thickening agents in the food industry [14]. Spent hop extracts have further shown beneficial effects on platelet and endothelial function, highlighting their potential in cardiovascular‐related applications [15].

The chemical composition of hot trub depends on the type of hop and on brewing conditions, including the beer style and processing parameters. Previous work has demonstrated the antioxidant potential of hot trub extracts from different beer types and the influence of ethanol‐water ratios on the yield of bioactive compounds [16, 17]. Ethanol–water mixtures are particularly suitable for extracting phenolic compounds and are preferred because they are less toxic than many organic solvents and generate less hazardous waste [18].

Cancer arises from genetic mutations that drive uncontrolled proliferation of tumor cells, leading to the formation of drug‐resistant tumors that can invade adjacent tissues and metastasize. Standard treatments such as surgery, radiotherapy, and chemotherapy are often limited by systemic toxicity and nonspecific effects on healthy cells. Nanotechnology offers a promising strategy to improve cancer therapy by enabling targeted delivery, sustained release, and reduced doses of chemotherapeutic agents [19].

Recent advances in nanomedicine have demonstrated the potential of rationally designed nanosystems to enable more effective and safer cancer treatment, including metal–organic framework‐coated nanosheets for chemo‐gene therapy [20], tumor microenvironment‐responsive nanoplatforms for multimodal imaging‐guided treatment [21], and carrier‐free prodrug nanoassemblies for breast cancer therapy [22, 23].

Zein nanoparticles have emerged as a promising nanocarrier for anticancer agents. Zein, a maize‐derived protein, is soluble in aqueous ethanol (55%–90%, v/v), biodegradable, biocompatible, low‐cost, and capable of self‐assembly and surface functionalization with both hydrophobic and hydrophilic ligands [24, 25]. Compared with synthetic polymeric and inorganic systems, zein is a natural, renewable protein with GRAS status [24, 25], thereby avoiding concerns regarding organic solvent residues and the long‐term accumulation of non‐degradable synthetic materials. However, zein nanoparticles are physically unstable at pH > 5.0 and under high‐temperature conditions, which limits their oral administration in the near‐neutral environment of the intestine [25, 26].

To overcome these limitations, zein nanoparticles are often complexed with natural biopolymers that enhance colloidal stability and bioavailability. Sodium caseinate, a naturally occurring protein, forms stable complexes with zein via electrostatic interactions, preventing aggregation and improving dispersibility and oral bioavailability [26, 27]. Chitosan, a cationic polysaccharide derived from chitin, is another widely used coating material; it is biodegradable, mucoadhesive, non‐toxic, and biocompatible, and can form bilayer complexes with zein through complex coacervation [28]. Chitosan‐coated zein nanoparticles show improved stability in acidic gastric conditions, prolonged release in the gastrointestinal tract, and enhanced resistance to aggregation due to combined steric and electrostatic repulsion [29, 30, 31].

Nanocarrier‐based delivery systems have emerged as promising platforms for cancer therapy, enabling improved delivery of therapeutic agents through rationally designed formulations [32]. In line with this, green‐synthesized nanoparticles can exhibit selective cytotoxicity against breast cancer cells while sparing normal cells, supporting the potential of green nanotechnological approaches in cancer therapy [33]. Among these, zein‐based nanocarriers loaded with natural polyphenols or other bioactives have demonstrated enhanced antiproliferative and pro‐apoptotic effects against breast cancer cells, underscoring the potential of this platform for cancer‐targeted delivery [34, 35]. More recently, primary studies have further confirmed the efficacy of zein‐based formulations against breast cancer, including glycyrrhizic acid‐loaded zein nanoparticles in MCF‐7 cells [36] and metformin‐loaded zein nanoparticles in an Ehrlich carcinoma breast cancer model [37]. Despite these advances, the valorization of brewing by‐products such as hot trub as a source of polyphenol‐rich extracts for nanoparticle‐mediated cancer therapy remains largely unexplored.

The main objective of this study was to extract bioactive compounds from hot trub by optimizing the ethanol‐water extraction process to reduce time and energy consumption while using a non‐toxic solvent. Furthermore, we prepared hot trub extract‐loaded zein nanoparticles (Z‐N), as well as chitosan‐coated (Z‐CHI‐N) and sodium caseinate‐coated (Z‐SC‐N) variants, and evaluated their effect on the viability of human breast cancer MCF‐7 cells.

## 2 Results and Discussion

### 2.1 Optimization of Hot Trub Extraction

The hot trub by‐product was dried in an oven for 48 h, affording a dry‐residue yield of 24%. The extracts from the dry residue were prepared using a 70:30 ethanol:water mixture, following the methodology of Tonin et al. [16]. The effects of time and temperature on extraction were evaluated by measuring antioxidant activity using DPPH, ABTS, and Fe (II) ion chelation assays, along with the quantification of phenolics and flavonoids. The results obtained under the specified conditions are presented in Table 1.

**TABLE 1:** Percentage of antioxidant activity (%AA) by DPPH, ABTS and Fe (II) ion chelating ability (CA) and phenolic (TPC) and flavonoids (TFC) quantification for the extracts of hot *trub*.

Time (h)	Temp. (°C)	%AA DPPH	%AA ABTS	%CA	TPC (mg GAE g^−1^)	TFC (mg RUE g^−1^)
0.69	45.0	70.66 ± 0.73^d^	99.42 ± 0.08^a, b^	94.99 ± 0.40^c^	4.42 ± 0.05^b, c^	2.05 ± 0.01^a, b^
4.0	30.0	74.51 ± 0.29^c^	89.06 ± 0.12^d, e^	87.08 ± 0.67^d^	4.26 ± 0.04^c^	2.03 ± 0.02^b^
4.0	60.0	82.86 ± 0.29^a^	99.74 ± 0.09^a^	96.33 ± 0.48^b, c^	4.77 ± 0.10^a^	2.05 ± 0.05^a, b^
12.0	23.8	62.73 ± 0.49^e^	84.37 ± 0.72^f^	96.93 ± 0.76^b, c^	3.97 ± 0.04^d^	1.80 ± 0.01^c^
12.0	45.0	78.31 ± 0.54^b^	88.34 ± 0.24^e^	96.77 ± 0.23^b, c^	4.26 ± 0.02^c^	2.04 ± 0.19^a, b^
12.0	45.0	77.19 ± 0.63^b, c^	83.63 ± 0.17^f^	95.96 ± 0.50^b, c^	4.29 ± 0.01^c^	1.97 ± 0.06^b^
12.0	45.0	77.17 ± 0.70^b, c^	88.23 ± 0.16^e^	96.49 ± 0.18^b, c^	4.65 ± 0.02^a, b^	2.19 ± 0.02^a^
12.0	66.2	81.58 ± 0.52^a^	99.43 ± 0.17^a, b^	82.98 ± 0.60^e^	4.35 ± 0.03^c^	2.01 ± 0.02^b^
20.0	30.0	78.62 ± 0.22^b^	97.41 ± 0.52^c^	98.14 ± 0.23^a^	4.33 ± 0.02^c^	2.02 ± 0.03^b^
20.0	60.0	82.57 ± 1.17^a^	97.95 ± 0.34^b, c^	72.88 ± 0.31^f^	4.49 ± 0.02^b, c^	2.19 ± 0.04^a^
23.3	45.0	65.01 ± 0.63^e^	90.23 ± 0.43^d^	86.09 ± 1.45^d^	4.30 ± 0.09^c^	2.06 ± 0.53^a^ ^, b^

Results expressed as mean ± standard deviation (*n* = 6). Values in the same column followed by different letters differ significantly (Tukey's test, *p* < 0.05).

Data analysis revealed that the extraction conditions of 4 and 20 h at 60°C, and 12 h at 66.2°C, resulted in the highest DPPH radical inhibition, which did not differ significantly (Tukey's test, *p* < 0.05). For the ABTS assay, the best results were observed at 0.69 h at 45°C, 4 h at 60°C, and 12 h at 66.2°C, again which did not differ significantly (Tukey's test, *p* < 0.05). For Fe (II) ion chelation ability (%CA), the highest chelation percentage was achieved at 20 h at 30°C.

Regarding total phenolic content (TPC), the optimal extraction conditions were 12 h at 45°C and 4 h at 60°C. The extraction conditions that favored flavonoid extraction were 0.69 h at 45°C, 4 h at 60°C, 12 h at 45°C, 20 h at 60°C, and 23.3 h at 45°C.

To provide a clearer understanding of how time and temperature affect the antioxidant activity, phenolic content, and flavonoid extraction from hot trub, theoretical models were generated based on the results from the experimental design shown in Table 1.

The models for the DPPH, ABTS assays, and phenolic and flavonoid quantification did not provide a good fit, yielding adjusted *R* ^2^ values of 0.4798, 0.7646, 0.4450, and 0.4013, respectively. However, the model for the chelating ability assay presented an *R* ^2^ of 0.9564, indicating a strong correlation. The ANOVA confirmed that all terms were significant (Table 2). The modeling of the experimental data was based on the following equation, where *t* is time and *T* is temperature:

(1) $$\begin{array}{ccc} \%\mathrm{CA}\; & = & 35.1814+4.0954t\;+2.0336T\;-0.0719\mathrm{tT}\;\\ & & \;-0.0521t^{2}-0.0164T^{2} \end{array}$$

**TABLE 2:** Analysis of variance for central composite rotatable design (CCRD) for antioxidant activity by chelating ability method.

Source of variation	df^a^	SS^b^	MS^c^	F value	F (Pr)	Significance
**Time (Q)**	1	376.94	376.94	128.70	0.0000	***
**Time (L)**	1	468.46	468.46	159.94	0.0000	***
**Temperature (L)**	1	967.81	967.81	330.43	0.0000	***
**Temperature (Q)**	1	451.18	451.18	154.04	0.0000	***
Time*Temperature	1	1785.84	1785.84	609.72	0.0000	***
**Residual**	60	4027.60	2.93			

Abbreviations; L, linear; Q, quadratic.

^a^ Degree of freedom;

^b^ Sum of squares;

^c^ Mean square; Pr(>F) (< 0.05 significant difference).

To explore the interactive effects of the operational parameters and determine the optimal levels of the variables, three‐dimensional and two‐dimensional surface plots (Figure 1a,b) were constructed based on Equation (1). The surface plots indicate that longer extraction times require lower temperatures to achieve optimal chelation. Three time–temperature combinations within the optimal range were identified for %CA: 4 h at 60°C, 12 h at 45°C, and 20 h at 30°C.

**FIGURE 1:**
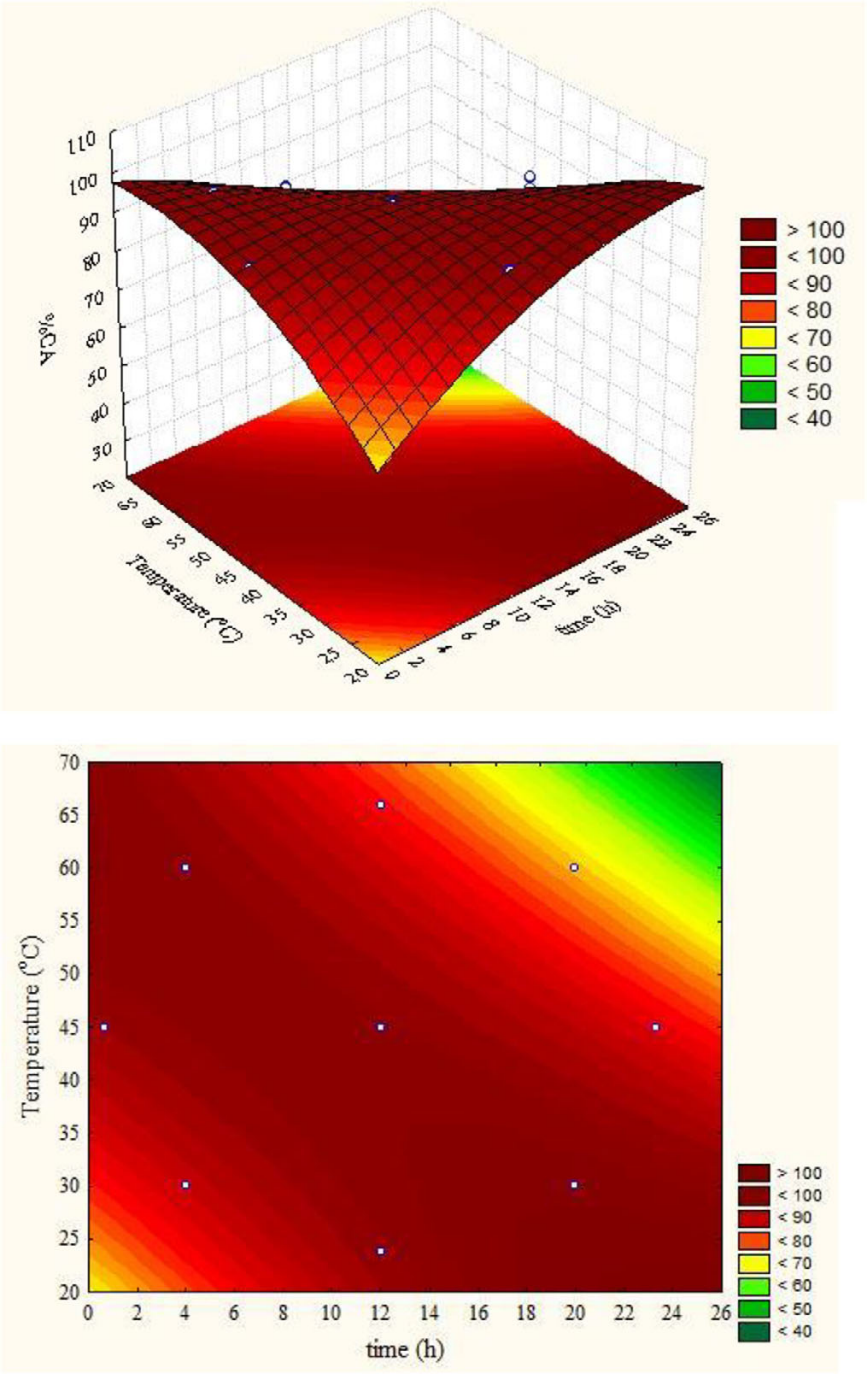
Response surface plots (a) 3D and (b) 2D showing the interactive effects of temperature and extraction time on the Fe(II) ion chelating ability (%CA) of hot trub extracts (original images by the authors).

In the context of antioxidant assays, it is important to recognize that different assays can reflect distinct mechanistic aspects of antioxidant activity. While DPPH and ABTS assess radical scavenging capacity, Fe (II) chelating ability evaluates metal‐binding capacity, which is a complementary antioxidant mechanism often linked to phenolic metal complexation. The strong model fit for chelating ability in our study (*R* ^2^ = 0.9564) suggests that extraction parameters influence not only radical scavenging but also metal ion interactions, which have implications for potential applications in food and cosmetic formulations.

Overall, the results indicate that both time and temperature influence the extraction of bioactive compounds from hot trub, with the best extraction conditions being 60°C for 4 h. It should be noted that the optimal extraction condition was selected on the basis of the measured responses, by comparing the mean values of the experimental runs with Tukey's test (*p* < 0.05), rather than on the predictions of the fitted regression models. Among the conditions evaluated, 4 h at 60°C simultaneously provided the highest DPPH and ABTS radical scavenging activities and the highest total phenolic content, together with a Fe(II) chelating ability close to the maximum observed, and was therefore considered the best multi‐response compromise. The regression models were used only for the response for which they provided an adequate fit (Fe(II) chelating ability, *R* ^2^ = 0.9564), from which the response surface plots were generated.

These findings suggest that the extraction of phenolic compounds from hot trub was favored by higher temperatures, up to a threshold beyond which thermal degradation may occur. Elevated temperatures likely enhance the permeability of plant tissue cells, increasing the solubility of compounds in the extraction system and boosting diffusion rates by weakening the interactions between phenolic compounds, proteins, and polysaccharides. Additionally, higher temperatures may reduce solvent viscosity and increase molecular mobility, thereby increasing mass transfer and improving extraction efficiency [38].

Our results are consistent with previous optimization studies demonstrating that hot trub is a significant source of phenolic compounds. Gandolpho et al. [17] applied ultrasound‐assisted extraction (UAE) to hot trub and optimized ethanol concentration, solid–liquid ratio, and extraction temperature through a central composite rotatable design (CCRD). Under the optimal conditions (58% ethanol, 1:32 solid:liquid ratio, 36°C, 30 min), the total phenolic content reached 7.57 ± 0.42 mg GAE g^−^^1^, confirming that hot trub can yield substantial amounts of phenolic compounds when appropriate extraction parameters are applied.

Although the TPC observed for the optimized extract in the present study (4.77 ± 0.10 mg GAE g^−^^1^, 70:30 ethanol:water) was lower than that reported by Gandolpho et al. [17], this difference may be attributed to the extraction technique and the parameter set applied. UAE enhances mass transfer by disrupting cell walls through acoustic cavitation, thereby improving extraction efficiency and phenolic yield, whereas the dynamic maceration approach used here relies on solvent penetration and thermal diffusion. In addition, the ethanol concentration employed (70%) exceeds the optimal range identified by Gandolpho et al. [17] (58%, with TPC decreasing approximately 37%), which may have further contributed to the lower yield. Differences in the raw material, such as the hop and malt varieties used in the brewing process, may also account for part of the observed variation.

Comparative studies have also indicated that hot trub extracts can contain a broad range of phenolic compounds with substantial antioxidant activity. Costa et al. [11] reported phenolic content ranging from approximately 8.2 to 13.4 mg GAE g–^1^ in different hot trub fractions (partitioned from the ethanolic extract prepared at room temperature using an ultrasonic bath) with antioxidant activity measured by both DPPH and ABTS methods. These values support the potential of hot trub as a source of antioxidant compounds and reinforce the concept that solvent systems, temperature and extraction conditions significantly influence quantifiable phenolic content and activity.

### 2.2 Identification of the Main Compounds in the Optimized Hot Trub Extract by Mass Spectrometry

The identification of phenolic compounds was performed by direct infusion ESI‐MS/MS, without prior chromatographic separation, as a rapid qualitative screening strategy rather than a quantitative determination. In this approach, matrix components may cause ion suppression or enhancement, potentially affecting the detection of low‐abundance compounds. However, the use of MRM mode, with compound‐specific precursor–product ion transitions, confers high specificity and minimizes the risk of false‐positive identifications, and each analyte was confirmed under two acquisition conditions.

The mass spectrometric profiling revealed a diverse array of phenolic compounds (Table 3): caffeic acid, caffeoyl‐hexoside, chrysin, p‐hydroxybenzoyl‐hexoside, and protocatechuic acid (Method 1); apigenin, catechin, catechin gallate, p‐coumaric acid, p‐hydroxybenzoic acid, isorhamnetin‐3‐O‐glucoside, kaempferol‐3‐coumaroylhexoside, luteolin‐7‐O‐glucoside, myricetin, quercetin‐3‐O‐hexoside, resveratrol, rutin, and syringic acid (Method 2). Notably, gallic acid, naringenin, and xanthohumol were confirmed by both analytical approaches (Table 3) [39, 40, 41, 42, 43, 44, 45, 46, 47, 48].

**TABLE 3:** Identification of compounds present in the extract of hot *trub* by the MRM method with collision energy of 15 eV (Method 1) and 30 eV (Method 2).

Analytes	MRM transition (*m/z*)	Collision energy (eV)	Detection	References
apigenin	269 > 151	30	√	Biesaga and Pyrzynska [39]
apigenin‐7‐glucoside	431 > 269	30	—	Pavlović et al. [40]
caffeic acid	179 > 161	15	√	Pavlović et al. [40]
caffeoyl‐hexoside	341 > 179	15	√	Pavlović et al. [40]
catechin	289 > 245	30	√	Yuzuak et al. [41]
catechin gallate	441 > 289	30	√	Yuzuak et al. [41]
chlorogenic acid	353 > 179	15	—	Pavlović et al. [40]
chrysin	253 > 209	15	√	Pavlović et al. [40]
*p*‐coumaric acid	163 > 119	30	√	Pavlović et al. [40]
coumaroyl‐hexoside	325 > 163	15	—	Pavlović et al. [40]
ellagic acid	301 > 257	15	—	Pavlović et al. [40]
ferulic acid	193 > 134	30	—	Sinosaki et al. [42]
feruloyl‐hexoside	355 > 193	15	—	Pavlović et al. [40]
gallic acid	169 > 125	15	√	Pavlović et al. [40]
	169 > 125	30	√	Pavlović et al. [40]
*p*‐hydroxybenzoic acid	137 > 93	30	√	Biesaga and Pyrzynska [39]
*p*‐hydroxybenzoyl‐hexoside	299 > 179	15	√	Pavlović et al. [40]
isorhamnetin‐3‐*O*‐glucoside	477 > 315	30	√	La Torre et al. [43]
kaempferol	285 > 161 285 > 257	15 30	‐ ‐	Pavlović et al. [40] March and Miao [44]
kaempferol‐3‐coumaroylhexoside	593 > 285	30	√	Pavlović et al. [40]
kaempferol‐hexoside	477 > 285	15	—	Pavlović et al. [40]
kaempferol‐3‐malonylhexoside	533 > 285	15	—	Pavlović et al. [40]
luteolin	285 > 133	15	—	Pavlović et al. [40]
luteolin‐7‐*O*‐glucoside	447 > 285	30	√	Pavlović et al. [40]
myricetin	317 > 151	30	√	Biesaga and Pyrzynska [39]
myricetin‐3‐O‐rutinoside	627 > 481	30	—	Lin et al. [45]
naringenin	271 > 177	15	√	Pavlović et al. [40]
	271 > 151	30	√	Biesaga and Pyrzynska [39]
naringin	579 > 271	30	—	Biesaga and Pyrzynska [39]
procyanidin B2	577 > 407	30	—	Xiao et al. [46]
protocatechuic acid	153 > 109	15	√	Pavlović et al. [40]
quercetin	301 > 179	30	—	Biesaga and Pyrzynska [39]
quercetin‐3‐*O*‐glucuronide	477 > 301	15	—	Pavlović et al. [40]
quercetin‐3‐*O*‐hexoside	463 > 301	30	√	Pavlović et al. [40]
quercetin‐3‐*O*‐xylopyranoside	433 > 301	15	—	Pavlović et al. [40]
resveratrol	227 > 185	30	√	Stella et al. [47]
rutin	609 > 301	30	√	Pavlović et al. [40]
sinapic acid	223 > 208	30	—	Sinosaki et al. [42]
syringic acid	197 > 153	30	√	Biesaga and Pyrzynska [39]
vanillic acid	167 > 123	30	—	Biesaga and Pyrzynska [39]
xanthohumol	353 > 233 353 > 119	15 30 15 30	√ √ √ √	Nikolic et al. [48]

Abbreviations: MRM, multiple reaction monitoring; – undetected; √ detected

Among these constituents, xanthohumol showed the highest relative signal intensity under both acquisition methods, suggesting that it is a major constituent of the extract; this assessment is based on relative signal intensity and does not constitute a quantitative determination. Xanthohumol is a prenylated chalcone uniquely abundant in Humulus lupulus that has been extensively studied for its potent biological activities, including antioxidant, anti‐inflammatory, neuroprotective, and anticancer effects. In particular, xanthohumol has exhibited inhibitory effects on carcinogenesis in drug‐resistant human colon carcinoma cells and induction of apoptosis in HT‐29 cells, reinforcing its chemopreventive potential [49, 50, 51, 52, 53, 54, 55].

The identification of a broad spectrum of phenolic acids and flavonoids in the present extract is consistent with previous characterizations of hot trub and related brewing by‐products. For instance, Costa et al. [11] reported similar phenolic profiles, including prenylflavonoids and hop bitter acids, in ethanol extracts of hot trub with significant antioxidant activity, suggesting that such compounds collectively contribute to radical scavenging and metal chelation. Additionally, Bravi et al. [55] identified dominant free phenolic acids such as caffeic, salicylic, and *p*‐coumaric acids in hot trub fractions from different beer styles, with observed variations in phenolic composition linked to brewing conditions and hop chemistry. Cortese et al. [56] quantified phenolic compounds in various types of craft beers, worts, and brewing by‐products, including hot trub, and identified a range of phenolic acids and flavonoids. Fărcaș et al. [57] quantified phenolic content and antioxidant activity in brewing by‑products, demonstrating that discharged waste contains appreciable levels of phenolic compounds with significant radical scavenging properties.

The identification of both common flavonoids (e.g., quercetin derivatives, luteolin glycosides) and hop‑specific prenylated flavonoids underscores the complex phytochemical nature of hot trub as a matrix rich in bioactive molecules. The presence of such molecules aligns with the notion that a significant proportion of hop‑derived phenolics and bitter acids is carried into the trub during wort boiling and protein precipitation, where they can be recovered and valorized [58]. This compositional richness reinforces the potential of hot trub not only as a source of antioxidants but also as a reservoir of high‑value compounds like xanthohumol with diverse biological functions.

Comparisons with broader brewing by‐product studies further illustrate the relevance of these findings. Hot trub is often underexploited relative to brewer's spent grain and spent yeast, despite containing unique phytochemicals that are less abundant in other residues. Its composition, encompassing polyphenols, bitter acids, and flavonoids, positions it as a promising feedstock for nutraceutical, cosmetic, and pharmaceutical applications, especially when optimized extraction conditions maximize recovery yields.

Importantly, the presence of flavonoids such as catechin and rutin, alongside phenolic acids like caffeic and syringic acids, supports the observed antioxidant potential measured via DPPH, ABTS, and Fe (II) chelation assays. These compounds are well‐documented in the literature for their radical scavenging and metal chelation capabilities, which are complementary mechanisms contributing to overall antioxidant activity [59, 60, 61].

Overall, the detailed mass spectrometric profiling presented here not only confirms the chemical complexity of hot trub extract but also aligns with contemporary studies highlighting its biotechnological potential. The identification of xanthohumol as a major constituent in combination with other phenolic compounds supports the notion that hot trub can serve as a valuable source of nutraceutical agents with antioxidant and potential therapeutic functions.

### 2.3 Photoprotective Activity of the Hot Trub Extract

The in vitro photoprotective activity of the optimized hot trub extract (EtOH:H_2_O 70:30 EtOH:H_2_O — 4 h at 60°C) was determined using the spectrophotometric method of Mansur et al. [62], which is widely used to estimate the sun protection factor (SPF) of plant extracts and experimental sunscreen formulations. In this study, the extract at 1000 µg mL^–^^1^ exhibited a significant photoprotective effect with an SPF value of 13.84 ± 0.93, exceeding the minimum SPF value (≥ 6) required by regulatory agencies [63] to be considered effective for cosmetic use. This finding demonstrates that the optimized extract possesses intrinsic UV‐absorbing capacity that could be relevant for dermocosmetic applications.

Natural extracts rich in phenolic compounds and flavonoids are increasingly investigated as potential photoprotective agents because these secondary metabolites absorb ultraviolet radiation and mitigate UV‐induced oxidative damage. Phenolic structures, including aromatic rings with conjugated double bonds, allow absorption of UV energy and contribute to radical scavenging, which is synergistic with photoprotection mechanisms. Reviews of phenolic photoprotective activity emphasize that the structural features of flavonoids and polyphenols facilitate UV absorption and attenuation of UV‐induced free radical formation, thereby enhancing SPF values in vitro [64, 65, 66].

Although the SPF value of the hot trub extract (13.84) was lower than that previously reported by the same research group for passion fruit seed extracts (18.22 and 16.68) [67], the value is still relevant among natural photoprotective agents. The comparatively lower SPF may reflect differences in phenolic profiles, extract composition, or concentration of UV‐absorbing moieties, underscoring the need for detailed characterization of extract constituents and their contributions to photoprotection. Multifactorial influences such as solvent polarity, phenolic structure, and extract concentration are known to impact SPF outcomes in spectrophotometric evaluations.

Therefore, the SPF results obtained with the hot trub extract not only demonstrate its standalone photoprotective potential but also highlight its potential for application in cosmetic formulations aimed at enhancing sun protection. These insights support further exploration of hot trub and similar brewery by‐product extracts as sustainable, plant‐derived photoprotective agents, with the potential to complement or partially substitute conventional UV filters in dermocosmetic products.

The photoprotective activity of the extract, evidenced by its in vitro SPF value, is consistent with its antioxidant capacity, since the phenolic constituents identified in the extract, including xanthohumol, are recognized contributors to both UV absorption and radical scavenging. This overlap is relevant to the therapeutic focus of the study, as oxidative stress is a common factor in UV‐induced skin damage and in carcinogenesis. In this context, the photoprotective and antioxidant properties of the extract align with its potential antitumor application, since the same phenolic pool that mitigates UV‐induced oxidative damage may also act through redox‐related mechanisms in cancer cells. It should be noted, however, that the present results do not demonstrate a chemopreventive effect, which would require dedicated in vivo studies.

### 2.4 Preparation, Characterization, and Encapsulation Efficiency of Zein‐Based NanoParticles

The choice of zein as the nanocarrier material was further supported by its suitability for the extract under study. Zein nanoparticles combine colloidal stability with a hydrophobic core well suited to the encapsulation of poorly water‐soluble polyphenols such as xanthohumol, the major compound identified in the hot trub extract (Table 3). In addition, the surface functionalization capacity of zein enabled the coating strategies evaluated here, in which chitosan and sodium caseinate were employed to widen the colloidal stability window of the formulations [68, 69, 70]. These characteristics position zein‐based systems as a sustainable alternative within the broader context of bio‐based nanomedicine.

Guided by these considerations, the preparation of the zein nanoparticles was systematically optimized, and the formulations were initially prepared at two different protein concentrations to determine the most suitable system for the encapsulation of the hot trub extract. The hydrodynamic size, polydispersity index (PDI), and zeta potential (ζ) for each sample are presented in Table 4. These physicochemical parameters provide critical insights into the colloidal stability of the nanoparticle suspensions.

**TABLE 4:** Characterization and encapsulation efficiency (EE) of zein nanoparticles at different zein: Extract (Z: E) concentrations.

C_Z_ (mg mL^−1^)	Hydrodynamic size (nm)	PDI (%)	ζ (mV)	EE (%)
10	152.97 ± 6.19^a^	21.83 ± 1.29^a^	+29.65 ± 3.26^a^	—
20	122.73 ± 1.26^b^	16.92 ± 1.02^b^	+33.87 ± 0.31^a^	—
**Z: E ratio (%)**				
0%	122.73 ± 1.26^b^	16.92 ± 1.02^a^	+33.87 ± 0.31^a^	—
20:1 (5%)	135.54 ± 3.99^a^ ^,b^	17.75 ± 1.16^a^	+25.93 ± 1.47^b^	n.d.
10:1 (10%)	150.50 ± 7.23^a^	19.87 ± 0.63^a^	+24.36 ± 0.86^b^	88.09 ± 3.65^a^
5:1 (20%)	142.54 ± 0.93^a^	16.40 ± 1.29^a^	+22.18 ± 0.52^c^	85.94 ± 0.90^a^

Abbreviation: n.d., not detected.

Results expressed as mean ± standard deviation (*n* = 2).

The PDI reflects the distribution of particle diameters; values ≤0.20 are commonly considered indicative of a homogeneous, monodisperse system. The zeta potential represents the surface charge of the particles, with absolute values greater than |30 mV| generally associated with electrostatically stabilized colloids due to repulsive forces that prevent aggregation [68].

The formulation containing 20 mg mL^–^^1^ zein was selected as the most suitable for further study, as it exhibited smaller particle size and lower PDI, suggesting a monodisperse colloidal system with reduced tendency for agglomeration. No significant difference (*p* < 0.05) was observed in zeta potential between the two zein concentrations tested, indicating both formulations maintain colloidal stability. Similar trends related to particle size reduction with increasing protein concentration have been reported for zein nanoparticle systems [71].

Using the optimized zein concentration, extract‐loaded zein nanoparticles were subsequently prepared using three mass ratios of zein to trub extract (20:1, 10:1, and 5:1). Upon encapsulation, increases in hydrodynamic diameter were observed at 10 % and 20 % extract loadings, likely due to the incorporation of higher amounts of phenolic constituents within the hydrophobic matrix. This trend has been observed in other bioactive‐loaded zein systems, in which core incorporation alters the internal structure and overall size of nanoparticles [72, 73]. In contrast, in some systems (e.g., curcumin‐loaded zein nanoparticles), hydrophobic bioactive compounds have been shown to influence particle compaction, resulting in decreased nanoparticle diameter due to enhanced hydrophobic interactions during antisolvent precipitation [69].

The PDI values for all extract‐loaded formulations remained within a narrow range, indicating that homogenized nanoparticle populations were maintained across different extract loadings. The positive ζ at pH ≈ 6 is consistent with the net positive surface charge expected below the isoelectric point of zein (pI ≈ 6.2) [74].

Encapsulation efficiency (EE) was negligible at the lowest extract ratio (5 %), whereas formulations containing 10 % and 20 % extract exhibited high EE values of 88.09 % and 85.94 %, respectively, without significant differences between them. The 10 % loading was selected for subsequent antitumor activity studies due to its comparatively higher zeta potential, which is often correlated with improved stability in biological environments. Prior studies employing resveratrol and chestnut fruit shell, cedar and sweetgum bark extracts in zein nanoparticles have demonstrated similarly high encapsulation efficiencies, underscoring the suitability of zein as a carrier for phenolic‐rich extracts [72, 75].

Zein–chitosan‐coated nanoparticles (Z‐CHI‐N) were characterized to understand the influence of polymer coating on colloidal properties (Table 5). For a starting zein concentration of 10 mg mL^–^^1^, only the 40:1 zein:chitosan ratio maintained comparable hydrodynamic size to uncoated particles; all other ratios yielded larger sizes, reflecting additional polymer layering. The high zeta potential observed across samples indicates effective coating and colloidal stability, in agreement with literature reports where chitosan coatings have enhanced the physicochemical robustness of zein nanoparticles [68].

**TABLE 5:** Characterization and encapsulation efficiency (EE) of zein nanoparticles coated with chitosan in different zein: Chitosan (Z: CHI) and zein: Extract (Z: E) mass ratios.

Z: CHI ratio	Hydrodynamic size (nm)	PDI (%)	ζ (mV)	EE (%)
C zein = 10 mg mL^−1^				
1:0	152.97 ± 6.19^c^	21.83 ± 1.29^b^	+29.65 ± 3.26^c^	—
10:1	244.92 ± 4.09^a^	25.95 ± 0.72^a^	+47.13 ± 1.17^a^	—
20:1	175.22 ± 2.78^b^	24.92 ± 0.51^a^	+46.83 ± 1.63^a^	—
40:1	150.63 ± 2.16^c^	23.83 ± 0.52^a^	+41.45 ± 0.35^b^	—
**C zein = 20 mg mL^−1^**				
1:0	122.73 ± 1.26^d^	16.92 ± 1.02^c^	+33.87 ± 0.31^c^	—
10:1	260.68 ± 5.84^a^	25.32 ± 0.40^a^	+47.85 ± 1.69^a^	—
20:1	197.69 ± 6.84^b^	24.12 ± 0.23^a,b^	+43.30 ± 0.62^b^	—
40:1	176.61 ± 3.70^c^	22.35 ± 0.71^b^	+43.98 ± 0.39^a,b^	—
**Z: E ratio (%)**				
1:0	150.63 ± 2.16^a^	23.83 ± 0.52^a^	+41.45 ± 0.35^a^	—
20:1 (5%)	136.99 ± 1.55^b^	24.35 ± 0.75^a^	+36.33 ± 0.95^b^	n.d.
10:1 (10%)	135.89 ± 2.18^b^	24.58 ± 0.31^a^	+37.52 ± 0.83^b^	83.51 ± 1.70^a^
5:1 (20%)	135.33 ± 1.56^b^	24.58 ± 0.57^a^	+31.60 ± 1.16^c^	85.05 ± 1.46^a^

Abbreviation: n.d., not detected.

Results expressed as mean ± standard deviation (*n* = 2).

When trub extract was encapsulated in the Z‐CHI‐N system, decreased hydrodynamic sizes were observed for all tested ratios, while PDI values remained unaltered. High encapsulation efficiency was maintained for both 10 % and 20 % extract loadings, with the 10 % formulation demonstrating the highest zeta potential and, by extension, greater colloidal stability (Table 5). The positive charge conferred by chitosan at neutral pH likely contributes to enhanced repulsive forces between particles, thereby improving dispersion stability [68].

For sodium caseinate‐coated zein nanoparticles (Z‐SC‐N), different zein:sodium caseinate ratios were investigated (Table 6). The 1:0.8 ratio yielded the smallest hydrodynamic diameter and PDI, indicative of an optimized coating that promotes stable structuring around the zein core. Notably, the zeta potential inverted from positive to negative after coating, confirming the successful adsorption of sodium caseinate on the nanoparticle surface. This inversion phenomenon has been reported previously for caseinate‐stabilized zein carriers, where the anionic protein provides steric and electrostatic barriers against aggregation [70, 76].

**TABLE 6:** Characterization and encapsulation efficiency (EE) of zein nanoparticles coated with sodium caseinate in different zein: Caseinate (Z: SC) and zein: Extract (Z: E) mass ratios.

Zein: SC ratio	Hydrodynamic size (nm)	PDI (%)	ζ (mV)	EE (%)
1:0	122.73 ± 1.26^d^	16.92 ± 1.02^a^	+33.87 ± 0.31^a^	—
1:0.6	221.50 ± 2.50^a^	15.00 ± 4.16^a^	−26.00 ± 0.37^c^	—
1:0.8	183.50 ± 2.84^c^	15.20 ± 5.14^a^	−27.70 ± 0.35^b^	—
1:1	210.70 ± 1.70^b^	9.90 ± 2.56^b^	−27.30 ± 0.42^b^	—
**Zein: Extract (%)**				
1:0 (0%)	183.5 ± 2.84^b^	15.20 ± 5.14^a,b^	−27.70 ± 0.35^a^	—
20:1 (5%)	237.80 ± 2.78^a^	15.37 ± 1.85^a,b^	−27.80 ± 1.50^a^	n.d.
10:1 (10%)	240.12 ± 2.60^a^	15.13 ± 0.88^a,b^	−28.48 ± 0.19^a^	90.70 ± 0.22^a^
5:1 (20%)	234.78 ± 5.37^a^	12.18 ± 2.07^b^	−28.65 ± 0.80^a^	52.52 ± 0.81^b^

Abbreviation: n.d., not detected.

Results expressed as mean ± standard deviation (*n* = 2).

Upon loading the trub extract into Z‐SC‐N systems at varying mass ratios (5 %, 10 %, 20 %), hydrodynamic sizes increased relative to the unloaded caseinate system, although differences among these conditions were not statistically significant. The highest encapsulation efficiency was achieved at the 10 % extract ratio, corresponding with PDI and zeta potential values consistent with a stable colloidal system suitable for downstream biological evaluation.

The preparation and characterization of the zein‐based nanoparticles successfully demonstrated their capacity to encapsulate bioactive compounds from hot trub extract. The optimization of the zein concentration and extract loading significantly influenced key physicochemical properties, such as particle size, PDI, and zeta potential, ensuring the formation of stable, homogeneous colloidal systems, and the coating with chitosan and sodium caseinate further enhanced their stability and encapsulation efficiency. Although the colloidal characterization indicates adequate physical stability of the formulations at the preparation stage, dedicated studies of colloidal stability under physiologically relevant pH and temperature conditions, as well as in vitro release kinetics, are required to support translational applications. Accordingly, further in‐depth characterization of the nanoparticles' stability over time, together with comprehensive in vitro testing of their biological activity, biocompatibility, and release profiles, is planned as future work.

### 2.5 In Vitro Cell Viability of Hot Trub Extract and Its Nanoparticle Formulations

The cell viability of the optimized hot trub extract on breast cancer cells (MCF‐7) were evaluated, with the results presented in Table 7. Asterisks indicate statistically significant reductions in absorbance compared with the negative control, highlighting the cytotoxic effects of the trub extract. After 24 h, only the concentration of 1000 µg mL^–^^1^ showed significant inhibition, with the mean absorbance lower than that of the negative control. After 48 h, concentrations of 100, 300, 400, 500, and 1000 µg mL^−^^1^ also demonstrated a decrease in cell viability. After 72 h, concentrations greater than or equal to 300 µg mL^–^^1^ exhibited significantly reduced cell viability. Specifically, the highest concentration (1000 µg mL^–^^1^) resulted in cell viabilities of 64.53% (24 h), 69.85% (48 h), and 50.01% (72 h), indicating that the extract reduced MCF‐7 cell viability in a concentration‐ and time‐dependent manner.

**TABLE 7:** Cell Viability (CV) percentage of MCF‐7 cells exposed to different concentrations of hot trub extract.

Concentration (µg mL^−1^)	24 h	48 h	72 h
CO‐	100.00	100.00	100.00
CO+	57.97*	41.09*	22.60*
5	90.70	114.23	86.78
10	100.00	87.11	100.51
50	93.02	88.51	105.68
100	89.48	71.04*	111.18
200	95.52	84.38	81.04
300	107.50	76.72*	73.04*
400	90.81	77.41*	68.87*
500	95.17	77.36*	61.85*
1000	64.53*	69.85*	50.01*

Values marked with an asterisk (*) differ significantly from the negative control (*p* < 0.05) according to Dunnett's test. CO‐: Negative control (untreated cells); CO+: Positive control.

Xanthohumol, identified as one of the major phenolic compounds in the trub extract, has been extensively studied for its anticancer properties, including apoptosis induction, inhibition of cell proliferation, and modulation of signaling pathways in a variety of cancer cell models. Previous work has demonstrated xanthohumol's ability to suppress proliferation in MCF‐7 cells and other cancer types in a dose‐dependent manner, supporting its relevance as a bioactive agent in the extract [77, 78, 79, 80, 81].

In addition to the intrinsic activity of xanthohumol, emerging evidence shows that zein‐based nanocarriers can enhance antitumor activity by improving cellular uptake, stability, and targeted delivery of therapeutic compounds. Zein nanoparticles have been increasingly recognized as promising platforms for drug delivery in cancer therapy due to their biodegradability, biocompatibility, low toxicity, and capacity to encapsulate hydrophobic compounds effectively. Their ability to modify surface properties and enhance drug loading has positioned zein nanocarriers as key candidates to improve the therapeutic index of encapsulated agents [24]. Building on these findings, we next evaluated the cell viability of the zein nanoparticles loaded with hot trub extract, comparing their performance with the free extract and empty nanoparticles.

Table 8 presents the cell viability results for zein nanoparticles, both loaded with and without trub extract, evaluated against MCF‐7 cells.

**TABLE 8:** Cell Viability (CV) percentage of MCF‐7 cells exposed to different concentrations of zein (Z), zein‐chitosan (Z‐CHI) and zein‐caseinate (Z‐SC) nanoparticles loaded with hot trub extract.

C (µg mL^−1^)	Z 0%			Z 10%			Z‐CHI 0%			Z‐CHI 10%			Z‐SC 0%			Z‐SC 10%		
	24 h	48 h	72 h	24 h	48 h	72 h	24 h	48 h	72 h	24 h	48 h	72 h	24 h	48 h	72 h	24 h	48 h	72 h
CO‐	100.00	100.00	100.00	100.00	100.00	100.00	100.00	100.00	100.00	100.00	100.00	100.00	100.00	100.00	100.00	100.00	100.00	100.00
CO+	54.20*	49.02*	47.93*	43.90*	44.70*	29.65*	53.40*	42.12*	29.06*	37.16*	40.30*	34.62*	56.05*	55.82*	29.79*	48.38*	40.93*	27.87*
5	122.27	80.24	111.35	116.30	58.11*	93.01	97.77	87.15	73.19*	86.46	83.08	87.45	110.51	75.54	81.50	100.09	68.08	87.68
10	114.18	89.30	114.08	104.56	62.68*	90.43	82.06*	95.68	75.38*	73.36*	79.63	87.99	104.46	74.57	75.59*	109.39	75.31	85.01
50	105.62	107.42	103.71	105.91	73.60	88.13	69.75*	99.53	73.47*	65.47*	85.24	91.48	104.67	77.93	86.35	106.95	73.86	93.67
100	103.40	80.79	99.34	91.08	68.71	78.49*	77.39*	78.33	76.13*	66.91*	66.59*	82.88	98.62	80.31	84.78	114.35	68.63	85.64
200	104.46	104.04	93.34	103.69	72.45	82.63	80.57*	85.18	87.18	71.82*	95.37	93.88	102.87	86.92	93.31	102.71	80.76	99.09
300	94.93	73.36*	91.92	105.04	61.02*	75.92*	85.35*	71.86*	73.06*	77.41	68.32	95.58	87.79	77.60	96.78	106.77	98.78	94.72
400	86.07	97.93	96.07	93.68	97.19	86.02	74.73*	110.41	74.49*	64.41*	95.26	96.51	93.74	134.28	89.50	101.81	127.55	102.32
500	100.99	100.87	76.75*	102.25	85.97	82.63	86.31*	91.46	63.17*	72.98*	81.14	90.55	101.80	132.23	81.76	119.31	120.13	93.31
1000	90.69	71.94*	60.70*	96.66	79.00	79.17*	97.03	86.59	58.66*	81.35	82.76	68.63*	117.09	135.48	112.93	116.34	128.29	100.49

Values marked with an asterisk (*) differ significantly from the negative control (*p* < 0.05) according to Dunnett's test. CO‐: Negative control (untreated cells); CO+: Positive control.

The zein nanoparticles loaded with trub extract (Z 10%) also reduced MCF‐7 cell viability, with significant decreases observed at 5 and 300 µg mL^–^^1^ after 48 h (cell viability of 58.11% and 61.02%, respectively) and at 100, 300, and 1000 µg mL^–^^1^ after 72 h (78.49%, 75.92%, and 79.17%, respectively). Notably, the encapsulated extract significantly reduced cell viability at 5 µg mL^–^^1^ (48 h) and 100 µg mL^–^^1^ (72 h), concentrations at which the free extract showed no significant effect (114.23% and 111.18%, respectively). At higher concentrations (≥300 µg mL^–^^1^), however, the free extract led to lower cell viability than the encapsulated system. These results indicate that encapsulation in zein nanoparticles did not enhance the overall effect of the extract, but extended its activity to lower and intermediate concentrations, which may reflect a more gradual exposure of the cells to the encapsulated bioactive compounds.

Chitosan‐coated zein nanoparticles ‐loaded with trub extract (Z‐CHI 10%) also reduced MCF‐7 cell viability, with significant decreases observed at 10, 50, 100, 200, 400, and 500 µg mL^–^^1^ after 24 h and at 100 µg mL^–^^1^ after 48 h; after 72 h, a significant reduction was observed only at 1000 µg mL^–^^1^ (68.63%). The responses of this formulation varied considerably across concentrations and time points, and the early effect observed at 24 h was not sustained at later time points, which precludes a clear time‐dependent trend and warrants cautious interpretation. Consistent with previous reports on curcumin‐ and berberine‐loaded zein‐chitosan nanoparticles, which showed increased cellular uptake and apoptosis in breast cancer cells [82], this coating strategy may favor the delivery of the encapsulated compounds. Sodium caseinate‐coated zein nanoparticles loaded with trub extract (Z‐SC 10%) promoted numerical reductions in MCF‐7 cell viability at 48 h (68%–81% in the 5–200 µg mL^–^^1^ range) and 72 h (85%–99% in the same range); however, none of these reductions reached statistical significance relative to the negative control. At 24 h, cell viability values above 100% were recorded across the tested concentrations; these values did not differ significantly from the control and were not sustained at later time points, indicating that they do not represent genuine proliferative effects. The numerical trends observed at intermediate concentrations, in the absence of significant effects at higher concentrations, suggest a modest response of this formulation under the tested conditions. Conclusions regarding a controlled‐release profile would require dedicated release kinetics studies, which are planned as future work.

The apparent increases in cell viability observed at 24 h are consistent with hormetic responses to polyphenol‐rich extracts or with nanoparticle interference in the MTT assay (e.g., interaction with formazan crystals or light scattering), rather than with genuine cell proliferation.

The studies collectively support the potential of zein‐based formulations to extend and modulate the activity of the extract at lower concentrations. This outcome are consistent with recent reports on zein‐based formulations against breast cancer, including glycyrrhizic acid‐loaded zein nanoparticles, which showed enhanced cytotoxicity and apoptosis induction in MCF‐7 cells [36], and metformin‐loaded zein nanoparticles, which demonstrated antitumor activity in an in vivo breast cancer model [37]. The data suggest that encapsulation extended the effect of the extract to lower concentrations rather than potentiating its overall activity. These findings support further investigation of zein‐based nanoparticle systems in cancer research, although additional optimization of the formulations and in vivo efficacy studies are still required.

The present study was designed as an in vitro investigation, and its scope should be considered when interpreting the results. The MTT assay evaluates the metabolic activity of viable cells and, as such, reflects an overall effect on cell viability without discriminating between cytostatic and cytocidal mechanisms. In addition, the biological evaluation was conducted in a single tumor cell line (MCF‐7), which corresponds to the luminal A molecular subtype of breast cancer (hormone receptor‐positive, generally less aggressive); therefore, the present results may not be representative of other breast cancer subtypes, such as HER2‐positive or triple‐negative phenotypes. The absence of a non‐tumorigenic counterpart also limits any inference regarding selectivity. These findings therefore provide preliminary in vitro evidence of the bioactivity of hot trub extract and its nanoparticle formulations against MCF‐7 cells, and further studies are warranted to elucidate the underlying mechanisms (e.g., apoptosis assays, cell cycle analysis, and evaluation of key signaling proteins), to assess selectivity in non‐tumorigenic cell lines, to evaluate the formulations in more complex biological models, and to test additional breast cancer cell lines representing different molecular subtypes (e.g., triple‐negative MDA‐MB‐231 and HER2‐positive models).

## 3 Conclusions

This study indicates that hot trub, a by‐product of the brewing industry, is a valuable source of bioactive compounds with significant antioxidant activity and in vitro effects on MCF‐7 cell viability. The optimization of the extraction process using a solvent mixture of ethanol and water allowed for the efficient recovery of a diverse range of phenolic compounds, including xanthohumol, phenolic acids, and flavonoids, all of which contribute to the bioactivity of the extract. The extract exhibited antioxidant and photoprotective properties, suggesting its potential for dermocosmetic applications. The encapsulation of the hot trub extract in zein‐based nanoparticles, both uncoated and coated with chitosan or sodium caseinate, enhanced the in vitro effect on MCF‐7 cell viability, and the coatings appeared to contribute to the stability and bioavailability of the nanoparticles. These findings suggest that zein‐based nanocarriers offer a promising platform for improving the delivery and in vitro bioactivity of the extract. However, before clinical applications can be considered, further studies are required, including mechanistic investigations (e.g., apoptosis and cell cycle analysis), long‐term stability and release profile assessments, safety and selectivity evaluation in non‐tumorigenic cell lines, and in vivo studies to confirm efficacy and biocompatibility. The ethanol–water solvent system employed is aligned with green chemistry principles, and regulatory aspects concerning extract standardization and contaminant control will be addressed during product development. From an industrial perspective, hot trub is generated in large quantities directly at the brewery site, and its recovery as a co‐product aligns with circular economy principles. The extraction process described here employs an ethanol–water solvent system at moderate temperature and does not require specialized equipment, which are favorable characteristics for scalability; however, technical and economic feasibility studies are required to establish the process at an industrial scale. Since the composition of hot trub may vary with the brewing process and hop variety, standardization of the extract based on the marker compounds identified in this study (e.g., xanthohumol, and total phenolic and flavonoid contents) will be essential to ensure batch‐to‐batch reproducibility and commercial applicability. This work underscores the biotechnological potential of hot trub as a sustainable source of bioactive agents with applications in the pharmaceutical and cosmetic industries.

## 4 Experimental Section

### 4.1 Sample Preparation

Two independent batches of craft beer were produced between May and August 2019, using 5.0 kg Pilsen malt, 0.86 kg Munich malt, and Bobek hops (20.0 g added 60 min before the end of the boil and 40.6 g at the start of boiling) as described by [16]. The hot trub obtained from both batches was pooled, dried in a forced‐air oven (Solab, model 102/480) at 40°C until a constant weight, crushed, and stored at −20°C until further analysis.

### 4.2 Optimization of Bioactive Compounds Extraction From Hot Trub

Extracts were prepared weighing 1.000 g of the dehydrated by‐product and mixed it with 50.0 mL of a 70:30 (v/v) ethanol:water solution (nominal concentration, C = 20 g L^−1^). The extraction was performed an orbital shaker at 150 rpm according to a central composite rotatable design (CCRD) (Table 9). The design comprised a 2^2^ full factorial (4 runs), 3 center points, and 4 axial points (α = ±1.41), totaling 11 experiments. Each of the 11 experimental runs was carried out in duplicate. The factors evaluated were extraction time and temperature. The extracts were filtered through filter paper and made up to a final volume of 50 mL with 70:30 (v/v) ethanol:water solution. The resulting solutions were stored in amber vials under refrigeration until further analysis. The response variables were antioxidant activity (DPPH, ABTS, and chelating ability), as well total phenolic and flavonoid contents. The optimized extract was concentrated by rotary evaporation and lyophilized (SL‐404, Solab).

**TABLE 9:** Variables and their levels employed in a central composite rotatable design (CCRD) for optimization of hot trub extracts.

Independent variables	Levels				
	−1.41	−1	0	+1	+1.41
**Temperature (°C)**	23.8	30	45	60	66.2
**Time (h)**	0.69	4	12	20	23.3

### 4.3 Determination of Antioxidant Activity and Quantification Bioactive Compounds

#### 4.3.1 DPPH Radical Scavenging Assay

The DPPH radical scavenging activity was determined in triplicate using the method of Brand–Williams et al. [83] as modified by Bressiani et al. [84]. Antioxidant activity was expressed as a percentage (%) in relation to the control (Equation 2).

(2) $$\%\mathrm{AA}=\frac{\left[A_{\mathrm{control}}-\left(A_{\mathrm{samp}\mathrm{le}}-\mathrm{Abla}\mathrm{nk}\right)\right]}{A_{\mathrm{control}}}\;$$

where *A*_control_ is the absorbance of the control (radical solution without extract); *A*_sample_ is the absorbance of the test sample; *A*_blank_ is the absorbance of the sample blank (accounting for the extract's intrinsic color).

#### 4.3.2 ABTS^+^ Radical Scavenging Assay

ABTS radical scavenging activity was determined in triplicate following the method of Rufino et al. [85], and antioxidant activity was also expressed as a percentage in relation to the control, using Equation (2).

#### 4.3.3 Fe (II) Ion Chelating Ability

The chelating ability of Fe (II) ions was determined in triplicate using the method described by Stookey [86] with modifications by Borosky et al. [87]. The percentage of chelating ability (%CA) was calculated using Equation (2).

#### 4.3.4 Total Phenolic Content (TPC)

TPC was determined in triplicate using the Folin–Ciocalteu reagent method, as described by Singleton and Rossi [88] as modified by dos Santos et al. [67]. Results were expressed in mg gallic acid equivalents (GAE) per g of sample, with GAE calculated from a standard curve (y = 0.0054x – 0.2067, *R* ^2^ = 0.998). The standard curve was prepared using gallic acid concentrations of 100, 120, 160, 200, 240, 280, 320, and 360 mg L^−1^.

#### 4.3.5 Total Flavonoid Content (TFC)

TFC was determined in triplicate using the method of Woisky and Salatino [89], with modifications by dos Santos et al. [67]. Results were expressed in mg rutin equivalents (RUE) per g of sample, calculated from a standard curve (y = 0.0079x – 0.0482, *R* ^2^ = 0.987). The standard curve was prepared using rutin concentrations of 12.5, 25, 50, and 100 mg L^−1^.

### 4.4 Identification of Bioactive Compounds by Mass Spectrometry—MRM Method

The optimized hot trub extract was diluted in HPLC‐grade methanol (Merck), filtered with a 0.45 µm syringe filter, and injected directly into a Quattro Premier XE Tandem Mass Spectrometer (Waters, Milford, MA, USA) with an electrospray ionization (ESI) source. Elution was performed with methanol containing 0.1% aqueous ammonia (NH_4_OH) at a flow rate of 200 µL min^−1^. Two different conditions were used for desolvation gas and source temperatures: Method 1 (200°C and 110°C) and Method 2 (250°C and 110°C). The electrospray source was operated in negative ionization mode (ESI(‐)) with capillary voltages of 2.0 kV (Method 1) and 2.5 kV (Method 2). Multiple reaction monitoring (MRM) mode was applied to identify precursor and product ions for each analyzed compound. The MRM transitions correspond to the characteristic fragmentation pathways of each compound class, and the precursor and product ions were established on the basis of the fragmentation patterns reported in the literature for the respective compounds [39, 40, 41, 42, 43, 44, 45, 46, 47, 48].

### 4.5 Preparation of Nanoparticles

#### 4.5.1 Preparation of Zein Nanoparticles Loaded With Trub Extract

Zein nanoparticles (Z‐N) were prepared by the antisolvent precipitation method, as described by Wang et al. [90], with modifications. Zein solutions (10 and 20 mg mL^−1^) were prepared in 70:30 (v/v) ethanol:water, stirred for one hour, and then 10 mL was added dropwise (1 mL min^−1^) to 20 mL ultrapure water. The solution was stirred for one hour, and ethanol was evaporated using a rotary evaporator. The resulting suspensions were centrifuged at 6000 rpm for 10 min, and the supernatant was stored in amber flasks under refrigeration. For zein‐extract formulations, different mass ratios of zein:extract 20:1 (5%), 10:1 (10%), and 5:1 (20%) were tested. The extract was incorporated into the zein solubilization step in hydroethanolic medium, and the remaining procedure was performed as described. The nanoparticles were prepared in two independent replicates.

#### 4.5.2 Preparation of Zein Nanoparticles Coated With Chitosan and Loaded With Trub Extract

Zein‐chitosan nanoparticles (Z‐CHI‐N) were prepared by dissolving chitosan (1.0 mg mL^–^^1^) in aqueous 1% (v/v) acetic acid under stirring for 6 h. Zein solutions (10 and 20 mg mL^−1^) were prepared in 70:30 (v/v) ethanol:water and stirred for one hour. For the 10 mg mL^–^^1^ zein solution, aliquots of 10.0, 5.0, and 2.5 mL of the chitosan solution were transferred to separate vessels and made up to a final volume of 20.0 mL with ultrapure water. In all cases, the zein solution was added dropwise to the chitosan solution at a flow rate of 1 mL min^–^^1^, under constant stirring. The same procedure was adopted for the 20 mg mL^–^^1^ zein solution, yielding zein:chitosan ratios of 10:1, 20:1, and 40:1, respectively. The mixture was stirred for an additional hour, then ethanol was evaporated using a rotary evaporator (IKA RV 05 basic), and the solution was centrifuged (6000 rpm, 10 min). The supernatant was collected and stored in an amber flask under refrigeration. [91]. Zein‐chitosan nanoparticles loaded with trub extract were prepared by adding the extract during zein solubilization, using zein:extract mass ratios of 20:1 (5%), 10:1 (10%), and 5:1 (20%). The nanoparticles were prepared in two independent replicates.

#### 4.5.3 Preparation of Zein Nanoparticles Coated With Sodium Caseinate and Loaded With Trub Extract

Zein‐caseinate nanoparticles (Z‐SC‐N) were prepared by adding 15.0 mL of a 20 mg mL^−1^ zein solution in 70:30 (v/v) ethanol:water dropwise at a flow 1 mL min^−1^, to 45.0 mL of sodium caseinate solution, under constant magnetic stirring at room temperature. Sodium caseinate solutions were prepared by dissolving 180, 240, and 300 mg of sodium caseinate in 45.0 mL of ultrapure water under stirring for 41 min, corresponding to zein:caseinate mass ratios of 1:0.6, 1:0.8, and 1:1, respectively. A formulation without sodium caseinate (1:0) was also prepared as a control, in which the zein solution was added dropwise to ultrapure water, which served as the antisolvent, under the same stirring conditions. The resulting mixture was stirred for 2 h, and ethanol was removed under reduced pressure using a rotary evaporator. The dispersion was then centrifuged at 6000 rpm for 10 min, and the supernatant was collected and stored in an amber flask under refrigeration [71, 76, 92]. Zein‐caseinate nanoparticles loaded with trub extract were prepared by adding the extract during zein solubilization in the hydroethanolic medium, at zein:extract mass ratios of 20:1 (5%), 10:1 (10%), and 5:1 (20%), following the same procedure described above. The nanoparticles were prepared in two independent replicates.

### 4.6 Characterization of Nanoparticles

The hydrodynamic size, polydispersity index, and zeta potential (ζ) of the nanoparticles were measured by dynamic light scattering (DLS) using a Litesizer^TM^ 500 (Anton Paar, Austria). Each sample was analyzed in triplicate.

### 4.7 Encapsulation Efficiency (EE)

Encapsulation efficiency was determined in duplicate following the method of Li et al. [92], with modifications. A standard curve of the trub extract was prepared at concentrations of 1000, 500, 250, 100, 50, 25, 10, and 5 µg mL^−1^ (y = 0.00167x + 0.01289; *R* ^2^ = 0.9994). Absorbance was measured at 295 nm using a UV–vis spectrophotometer (Agilent Cary 60). Nanoparticle samples were diluted in ethanol, vortexed for 1 min, and centrifuged for 10 min. Encapsulation efficiency was calculated by subtracting the absorbance of the nanoparticle without extract from the total absorbance.

### 4.8 Photoprotective Activity of Hot Trub Extract

The SPF was determined using an in vitro spectrophotometric method. The optimized hot trub extract was dissolved at 1000 µg mL^–^^1^, and the absorbance of the solution was recorded across the UV range of 290–320 nm at 5 nm intervals using a UV–vis spectrophotometer (Agilent Cary 60). The SPF value was calculated from Equation (3), according to the method of Mansur et al. [62], which applies the erythemal efficiency and solar intensity spectra [EE(λ) × I(λ)]. The solution was prepared in duplicate, and absorbance readings were performed in triplicate.

(3) $$\mathrm{SPF}=CF.\sum_{290}^{320}EE\left(\lambda \right).I\left(\lambda \right).\left|\left(\lambda \right)\right|$$

where CF is the correction factor (10), EE(*λ*) is the erythemal efficiency spectrum, I(*λ*) is the solar intensity spectrum, and abs(*λ*) is the absorbance of the solution. The values of EE(*λ*) × I(*λ*) are constant.

### 4.9 In Vitro Cell Viability Assay (MTT)

The cell viability of the hot trub extract and nanoparticles loaded with trub extract was evaluated against the MCF‐7 human breast cancer cell line, provided by the Cell Bank of Rio de Janeiro, Brazil (BCRJ Code: 0162). Because no RRID is available for this cell line, detailed information regarding the origin and maintenance of the cell lines is provided to ensure reproducibility. The samples were diluted in culture medium supplemented with fetal bovine serum at concentrations of 5, 10, 50, 100, 200, 300, 400, 500, and 1000 µg mL^−1^. The assay was conducted as described by Mosmann [93] as modified by dos Santos et al. [67]. Cell viability (CV) was calculated by comparing the treatment absorbance to the negative control absorbance.

### 4.10 Statistical Analyses

Statistical analyses were performed using Statistica 8.0 software (StatSoft Inc., Tulsa, OK, USA). Results were expressed as mean ± standard deviation (SD). Antioxidant activity assays and bioactive compound quantification were carried out with extracts prepared in duplicate, and all readings were performed in triplicate. Nanoparticles were prepared in duplicate, and their measurements (hydrodynamic size, PDI, zeta potential, and encapsulation efficiency) were performed in triplicate. Data from the central composite rotatable design (CCRD 2^2^) were evaluated by analysis of variance (ANOVA), and a second‐order polynomial regression model was fitted to the experimental data, with coefficients estimated by ANOVA; model adequacy was verified by the coefficient of determination (*R* ^2^). Three‐dimensional response surface plots were generated from the fitted model. Nanoparticle data were analyzed by Tukey's test at a significance level of *p* < 0.05, which corrects for multiple pairwise comparisons. Cell viability data (*n* = 3) were analyzed by one‐way ANOVA followed by Dunnett's test (*p* < 0.05) using Action Stat software; for each time point, all tested concentrations were compared with the respective negative control, and Dunnett's test inherently controls the family‐wise error rate for multiple comparisons against a single control group. Since the study was designed as a bioactivity screening, comparisons between formulations were descriptive, and no claim of statistical superiority of one coating over another is made.

## Author Contributions

**Lilian Tatiani Dusman Tonin**: conceptualization, investigation, funding acquisition, writing – original draft, methodology, writing – review and editing, data curation, supervision. **Isabella Salvat Luccas**: investigation, methodology. **Gabriela Barbosa da Silva**: investigation, methodology. **Giovanna Martins Domingos**: investigation, methodology. **Grazielle Jesus dos Santos**: investigation, methodology, data curation. **Elisângela Düsman**: conceptualization, investigation, writing – original draft, methodology, data curation. **Ana Beatriz Sousa Santana**: investigation, methodology. **Patricia Aline Bressiani**: investigation, methodology. **Ágatha Giovanna Fragoso**: investigation, methodology. **Angélica Priscila Parussolo Tonin**: investigation, methodology, data curation. **Eduardo César Meurer**: investigation, writing – original draft, methodology, data curation.

## Funding

This work was supported by Coordenação de Aperfeiçoamento de Pessoal de Nível Superior (CAPES) (Finance code 001).

## Conflicts of Interest

The authors declare no conflicts of interest.

## Data Availability

The data that support the findings of this study are available from the corresponding author upon reasonable request.

## References

[1] A. Phaniendra , D. B. Jestadi , and L. Periyasamy , “Free Radicals: Properties, Sources, Targets, and Their Implication in Various Diseases,” Indian Journal of Clinical Biochemistry 30 (2015): 11–26. 10.1007/s12291-014-0446-0 PMC4310837 25646037

[2] P. Di Donato , V. Taurisano , G. Tommonaro , et al., “Biological Properties of Polyphenols Extracts From Agro Industry's Wastes,” Waste and Biomass Valorization 9 (2018): 1567–1578.

[3] J. Hubert , A. Angelis , N. Aligiannis , and J. H. R. Skaltsounis , “In Vitro Dermocosmetic Evaluation of Bark Extracts From Common Temperate Trees,” Planta Medica 82 (2016): 1351–1358. 10.1055/s-0042-110180 27352384

[4] H. I. M. Amin , A. H. Hamadamin , K. D. Issa , et al., “Synthesis of Copper Oxide Nanoparticles From Ixiolirion Tataricum: Assessment of Their Antimicrobial, Antioxidant, and Enzyme Inhibitory Activity,” Inorganic Chemistry Communications 184 (2026): 116024.

[5] BRASIL. Ministério da Agricultura “Pecuária e Abastecimento. Anuário da Cerveja 2024: Ano de Referência 2023. Ministério da Agricultura e Pecuária,” in Secretaria de Defesa Agropecuária (MAPA/SDA, 2024).

[6] T. R. S. Mathias , P. P. M. Mello , and E. F. C. Sérvulo , “Solid Wastes in Brewing Process: A Review,” Journal of Brewing and Distilling 5 (2014): 1–9.

[7] N. Huige , “Brewery by‐products and Effluents,” in Handbook, Brewing, 2nd ed., ed. F. G. Priest and G. G. Stewart (Taylor & Francis Group, 2006), 656–707.

[8] S. I. Mussato , “Chapter 16: Biotechnological Potential of Brewing Industry By‐Products,” in Biotechnology for Agro‐Industrial Residues Utilisation, ed. P. S. Nigam and A. Pandey (2009).

[9] G. T. Eyres and J. P. Dufour , “Hop Essential Oil: Analysis, Chemical Composition and Odor Characteristics,” in Beer in Health and Disease Prevention, ed. V. R. Preedy , (Academic Press, 2009), 239–254.

[10] L. Bocquet , S. Sahpaz , J. L. Hilbert , C. Rambaud , and C. Rivière , “ *Humulus Lupulus* L., a Very Popular Beer Ingredient and Medicinal Plant: Overview of Its Phytochemistry, Its Bioactivity, and Its Biotechnology,” Phytochemical Reviews 17 (2018): 1047–1090.

[11] F. S. F. Costa , T. R. Amparo , J. B. Seibert , et al., “Reuse of Hot Trub as an Active Ingredient With Antioxidant and Antimicrobial Potential,” Waste and Biomass Valorization 12 (2021): 2037–2047.

[12] M. Tatullo , G. M. Simone , F. Tarullo , et al., “Antioxidant and Antitumor Activity of a Bioactive Polyphenolic Fraction Isolated From the Brewing Process,” Scientific Reports 6 (2016): 36042. 10.1038/srep36042 PMC5081531 27786308

[13] S. Bedini , G. Flamini , J. Girardi , F. Cosci , and B. Conti , “Not Just for Beer: Evaluation of Spent Hops (*Humulus lupulus* L.) as a Source of Eco‐friendly Repellents for Insect Pests of Stored Foods,” Journal of Pest Science 88 (2015): 583–592.

[14] A. Oosterveld , A. G. J. Voragen , and H. A. Schols , “Characterization of Hop Pectins Shows the Presence of an Arabinogalactan‐protein,” Carbohydrate Polymers 49 (2002): 407–413.

[15] B. Luzak , J. Golanski , T. Przygodzki , et al., “Extract From Spent hop (*Humulus lupulus* L.) Reduces Blood Platelet Aggregation and Improves Anticoagulant Activity of human Endothelial Cells In Vitro,” Journal of Functional Foods 22 (2016): 257–269.

[16] L. T. D. Tonin , A. C. R. Aranha , D. A. Nardino , V. S. C. O. Trindade , I. S. Lucas , and N. L. Gatz , “Valorização de Resíduos Da Produção de Cerveja como Fonte de Compostos Bioativos Antioxidantes,” Exatas Online 12 (2021): 38–51.

[17] B. C. G. Gandolpho , A. R. Almeida , G. M. Gandolpho , et al., “Optimization of Brewing Waste's (trub) Phenolic Compounds Extraction by Ultrasound Assisted Using Response Surface Methodology,” Química Nova 44 (2021): 478–483.

[18] J. Shi , J. Yu , M. Bryan , and Y. Wu , “Optimization of the Extraction of Polyphenols From Grape Seed Meal by Aqueous Ethanol Solution,” Journal of Food, Agriculture, and Environment 1 (2003): 42–47.

[19] L. Zeng , B. H. J. Gowda , M. G. Ahmed , et al., “Advancements in Nanoparticle‐based Treatment Approaches for Skin Cancer Therapy,” Molecular Cancer 22 (2023): 1–50. 10.1186/s12943-022-01708-4 PMC9835394 36635761

[20] Y. Nie , D. Li , Y. Peng , et al., “Metal Organic Framework Coated MnO_2_ Nanosheets Delivering Doxorubicin and Self‐activated DNAzyme for Chemo‐gene Combinatorial Treatment of Cancer,” International Journal of Pharmaceutics 585 (2020): 119513. 10.1016/j.ijpharm.2020.119513 32526334

[21] Y. Wang , Y. Xu , J. Song , et al., “Tumor Cell‐Targeting and Tumor Microenvironment‐Responsive Nanoplatforms for the Multimodal Imaging‐Guided Photodynamic/Photothermal/Chemodynamic Treatment of Cervical Cancer,” International Journal of Nanomedicine 19 (2024): 5837–5858. 10.2147/IJN.S466042 PMC11182360 38887692

[22] C. Feng , Y. Wang , J. Xu , et al., “Precisely Tailoring Molecular Structure of Doxorubicin Prodrugs to Enable Stable Nanoassembly, Rapid Activation, and Potent Antitumor Effect,” Pharmaceutics 16 (2024): 1582. 10.3390/pharmaceutics16121582 PMC11679313 39771561

[23] H. Yuan , Y. Chen , Y. Hu , et al., “Disulfide Bond‐driven Nanoassembly of Lipophilic Epirubicin Prodrugs for Breast Cancer Therapy,” Journal of Pharmaceutical Investigation 55 (2025): 889–902.

[24] M. S. Khan , M. Fatima , G. Gupta , S. Md , and P. J. Kesharwani , “Recent Progress in Zein Nanoparticles: A Promising Approach for Advancing Cancer Therapy,” Drug Delivery Science and Technology 97 (2024): 105770.

[25] H. Yu , X. Ge , J. Kong , et al., “Development of Alantolactone‐loaded Zein and Shellac Nanoparticles for Controlled Release in Simulated Gastrointestinal Digestion,” Journal of Food Engineering 358 (2023): 111664.

[26] X. Yan , M. Li , X. Xu , X. Liu , and F. Liu , “Zein‐based Nano‐delivery Systems for Encapsulation and Protection of Hydrophobic Bioactives: A Review,” Frontiers in Nutrition 9 (2022): 1–16. 10.3389/fnut.2022.999373 PMC9554640 36245539

[27] F. Li , Y. Chen , S. Liu , et al., “Size‐controlled Fabrication of Zein Nano/Microparticles by Modified Anti‐solvent Precipitation With/Without Sodium Caseinate,” International Journal of Nanomedicine 12 (2017): 8197–8209. 10.2147/IJN.S143733 PMC5689028 29184408

[28] L. Ma , C. R. Su , S. Y. Li , S. He , A. Nag , and Y. Yuan , “Co‐delivery of Curcumin and Quercetin in the Bilayer Structure Based on Complex Coacervation,” Food Hydrocolloids 144 (2023): 108907.

[29] J. Liu , H. Yu , J. Kong , et al., “Preparation, Characterization, Stability, and Controlled Release of Chitosan‐coated Zein/Shellac Nanoparticles for the Delivery of Quercetin,” Food Chemistry 444 (2024): 138634. 10.1016/j.foodchem.2024.138634 38330608

[30] Z. Shariatinia , “Pharmaceutical Applications of Chitosan,” Advances in Colloid and Interface Science 263 (2019): 131–194. 10.1016/j.cis.2018.11.008 30530176

[31] Y. Cao , Y. Yang , Z. Liang , et al., “Synthesis of Ganoderic Acids Loaded Zein‐Chitosan Nanoparticles and Evaluation of Their Hepatoprotective Effect on Mice Given Excessive Alcohol,” Foods 13 (2024): 2760. 10.3390/foods13172760 PMC11394847 39272525

[32] H. O. Othman , D. S. Ali , and R. O. Hassan , “AI‐Nanotechnology Synergy: Revolutionizing Precision Drug Delivery Systems: A Comprehensive Review,” Analytical Chemistry Letters 16 (2026): 95–115.

[33] K. D. Issa , H. O. Othman , H. I. M. Amin , et al., “Sustainable Antimicrobial and Anticancer Agents: Eco‐Friendly Synthesis of Copper Nanoparticles Using Biebersteinia Multifida DC,” Chemistry & Biodiversity 22 (2025): e202402612. 10.1002/cbdv.202402612 40262138

[34] H. I. Kutbi , A. K. Kammoun , and D. F. El‐Telbany , “Amelioration of Pterostilbene Antiproliferative, Proapoptotic, and Oxidant Potentials in Human Breast Cancer MCF7 Cells Using Zein Nanocomposites,” International Journal of Nanomedicine 16 (2021): 3059–3071. 10.2147/IJN.S303975 PMC8090986 33953555

[35] H. M. Alharbi , T. Alqahtani , N. A. Alqalawi , et al., “Exploring the Potential of Zein Nanoparticles in Personalised Cancer Therapy, Highlighting Their Various Methodologies, Applications and Challenges,” Journal of Cellular and Molecular Medicine 29 (2025): e70752. 10.1111/jcmm.70752 PMC12343330 40797083

[36] A. K. Srivastav , P. K. Rajput , J. Jaiswal , et al., “ *In Vitro* and *In Silico* Investigation of Glycyrrhizic Acid Encapsulated Zein Nanoparticles: A Synergistic Targeted Drug Delivery Approach for Breast Cancer,” International Journal of Biological Macromolecules 266 (2024): 131368. 10.1016/j.ijbiomac.2024.131368 38580025

[37] Y. Elmahboub , R. Albash , M. M. William , et al., “Metformin‐Loaded Zein Polymeric Nanoparticles to Augment Antitumor Activity Against Ehrlich Carcinoma via Activation of AMPK Pathway: D‐Optimal Design Optimization, *In Vitro* Characterization, and *In Vivo* Study,” Molecules 29 (2024): 1614. 10.3390/molecules29071614 PMC11013883 38611893

[38] M. Bachir bey , L. Meziant , Y. Benchikh , and H. Louaileche , “Deployment of Response Surface Methodology to Optimize Recovery of Dark Fresh Fig (*Ficus carica* L., var.Azenjar) Total Phenolic Compounds and Antioxidant Activity,” Food Chemistry 162 (2014): 277–282. 10.1016/j.foodchem.2014.04.054 24874388

[39] M. Biesaga and K. Pyrzynska , “Liquid Chromatography/Tandem Mass Spectrometry Studies of the Phenolic Compounds in Honey,” Journal of Chromatography A 1216 (2009): 6620–6626. 10.1016/j.chroma.2009.07.066 19691961

[40] A. V. Pavlović , D. Č. Dabić , N. M. Momirović , et al., “Chemical Composition of Two Different Extracts of Berries Harvested in Serbia,” Journal of Agricultural and Food Chemistry 61 (2013): 4188–4194. 10.1021/jf400607f 23600608

[41] S. Yuzuak , J. Ballington , and D. Y. Xie , “HPLC‐qTOF‐MS/MS‐Based Profiling of flavan‐3‐ols and Dimeric Proanthocyanidins in Berries of Two Muscadine Grape Hybrids FLH 13–11 and FLH 17–66,” Metabolites 8 (2018): 1–24. 10.3390/metabo8040057 PMC6316709 30261603

[42] N. B. M. Sinosaki , A. P. P. Tonin , M. A. S. Ribeiro , et al., “Structural Study of Phenolic Acids by Triple Quadrupole Mass Spectrometry With Electrospray Ionization in Negative Mode and H/D Isotopic Exchange,” Journal of the Brazilian Chemical Society 31 (2020): 402–408.

[43] G. L. La Torre , M. Saitta , F. Vilasi , T. Pellicanò , and G. Dugo , “Direct Determination of Phenolic Compounds in Sicilian Wines by Liquid Chromatography With PDA and MS Detection,” Food Chemistry 94 (2006): 640–650.

[44] R. E. March and X. S. Miao , “A Fragmentation Study of Kaempferol Using Electrospray Quadrupole Time‐of‐flight Mass Spectrometry at High Mass Resolution,” International Journal of Mass Spectrometry 231 (2004): 157–167.

[45] L. Z. Lin , P. Chen , M. Ozcan , and J. M. Harnly , “Chromatographic Profiles and Identification of New Phenolic Components of *Ginkgo Biloba* Leaves and Selected Products,” Journal of Agricultural and Food Chemistry 56 (2008): 6671–6679. 10.1021/jf800488x PMC3762682 18598036

[46] Y. Xiao , Z. Hu , Z. Yin , et al., “Profiling and Distribution of Metabolites of Procyanidin B2 in Mice by UPLC‐DAD‐ESI‐IT‐TOF‐MSn Technique,” Frontiers in Pharmacology 8 (2017): 1–12. 10.3389/fphar.2017.00231 PMC5415559 28522973

[47] L. Stella , M. de Rosso , A. Panighel , A. D. Vedova , R. Flamini , and P. Traldi , “Collisionally Induced Fragmentation of [M‐H]^−^ Species of Resveratrol and Piceatannol Investigated by Deuterium Labelling and Accurate Mass Measurements,” Rapid Communications in Mass Spectrometry 22 (2008): 3867–3872. 10.1002/rcm.3811 18980255

[48] D. Nikolic , Y. Li , L. R. Chadwick , G. F. Pauli , and R. B. van Breemen , “Metabolism of Xanthohumol and Isoxanthohumol, Prenylated Flavonoids From Hops (*Humulus lupulus* L.), by human Liver Microsomes,” Journal of Mass Spectrometry 40 (2005): 289–299. 10.1002/jms.753 15712367

[49] X. Liu , L. J. An , Y. Li , et al., “Xanthohumol Chalcone Acts as a Powerful Inhibitor of Carcinogenesis in Drug‐resistant human Colon Carcinoma and These Effects Are Mediated via G2/M Phase Cell Cycle Arrest, Activation of Apoptotic Pathways, Caspase Activation, and Targeting Ras /MEK/ERK pa,” Journal of Buon 24 (2019): 2442–2447. 31983118

[50] C. Jiang , N. Xie , T. Sun , W. Ma , B. Zhang , and W. Li , “Xanthohumol Inhibits TGFβ1‐induced Cardiac Fibroblasts Activation via Mediating PTEN/Akt/mTOR Signaling Pathway,” Drug Design, Development, and Therapy 14 (2020): 5431–5439. 10.2147/DDDT.S282206 PMC7732164 33324040

[51] M. Liu , H. Yin , X. Qian , J. Dong , Z. Qian , and J. Miao , “Xanthohumol, a Prenylated Chalcone From Hops, Inhibits the Viability and Stemness of Doxorubicin Resistant MCF‐7/ADR Cells,” Molecules 22 (2017): 36. 10.3390/molecules22010036 PMC6155764 28036030

[52] T. Yoshimaru , M. Komatsu , E. Tashiro , et al., “Xanthohumol Suppresses Oestrogen‐signalling in Breast Cancer Through the Inhibition of BIG3‐PHB2 Interactions,” Scientific Reports 4 (2014): 7355. 10.1038/srep07355 PMC4258681 25483453

[53] K. Zenger , S. Dutta , H. Wolff , M. G. Genton , and B. Kraus , “ *In Vitro* Structure Toxicity Relationship of Chalcones in Human Hepatic Stellate Cells,” Toxicology 336 (2015): 26–33. 10.1016/j.tox.2015.07.012 26201061

[54] X. Wang , S. L. Ho , C. Y. Poon , T. Yan , H. W. Li , and M. S. Wong , “Amyloid‐β Aggregation Inhibitory and Neuroprotective Effects of Xanthohumol and Its Derivatives for Alzheimer's Diseases,” Current Alzheimer Research 16 (2019): 836–842. 10.2174/1567205016666190827123222 31453789

[55] E. Bravi , G. D. Francesco , V. Sileoni , G. Perretti , F. Galgano , and O. Marconi , “Brewing by‐Product Upcycling Potential: Nutritionally Valuable Compounds and Antioxidant Activity Evaluation,” Antioxidants 10 (2021): 1–18. 10.3390/antiox10020165 PMC7911235 33499399

[56] M. Cortese , M. R. Gigliobianco , D. V. Peregrina , G. Sagratini , R. Censi , and P. Di Martino , “Quantification of Phenolic Compounds in Different Types of Crafts Beers, Worts, Starting, and Spent Ingredients by Liquid Chromatography‐tandem Mass Spectrometry,” Journal of Chromatography A 1612 (2020): 1–11. 10.1016/j.chroma.2019.460622 31653471

[57] A. Fărcaş , M. Tofană , S. Socaci , S. Scrob , L. Salanţă , and A. Borşa , “Preliminary Study on Antioxidant Activity and Polyphenols Content in Discharged Waste From Beer Production,” Journal of Agroalimentary Processes and Technologies 19 (2013): 319–324.

[58] K. F. C. Silva , M. M. Strieder , M. B. C. Pinto , M. A. Rostagno , and M. D. Hubinger , “Processing Strategies for Extraction and Concentration of Bitter Acids and Polyphenols From Brewing By‐Products: A Comprehensive Review,” Processes 11 (2023): 921.

[59] M. Grzesik , K. Naparło , G. Bartosz , and I. Sadowska‐Bartosz , “Antioxidant Properties of Catechins: Comparison With Other Antioxidants,” Food Chemistry 241 (2018): 480–492. 10.1016/j.foodchem.2017.08.117 28958556

[60] C. Srinivasulu , M. Ramgopal , G. Ramanjaneyulu , C. M. Anuradha , and C. S. Kumar , “Syringic Acid (SA) ‒ A Review of Its Occurrence, Biosynthesis, Pharmacological, and Industrial Importance,” Biomedicine & Pharmacotherapy 108 (2018): 547–557. 10.1016/j.biopha.2018.09.069 30243088

[61] E. Girsang , I. N. E. Lister , C. N. Ginting , et al., “Antioxidant and Antiaging Activity of Rutin and Caffeic Acid,” Pharmaciana 10 (2020): 147–156.

[62] J. D. S. Mansur , M. N. R. Breder , M. C. A. Mansur , and R. D. Azulay , “Determinação do Fator de Proteção Solar por Espectrofotometria,” Anais Brasileiros de Dermatologia 61 (1986): 121–124.

[63] BRASIL. Ministério da Saúde “Agência Nacional de Vigilância Sanitária. Resolução RDC no 30, de 01 de junho de 2012,” Diário Oficial da União, Brasília, DF, (2012).

[64] L. Li , L. Chong , T. Huang , Y. Ma , Y. Li , and H. Ding , “Natural Products and Extracts From Plants as Natural UV Filters for Sunscreens: A Review,” Animal Model and Experimental Medicine 6 (2023): 183–195. 10.1002/ame2.12295 PMC10272908 36536536

[65] J. Milutinov , N. Pavlović , D. Ćirin , M. Atanacković Krstonošić , and V. Krstonošić , “The Potential of Natural Compounds in UV Protection Products,” Molecules 29 (2024): 5409. 10.3390/molecules29225409 PMC11597743 39598798

[66] N. Saewan and A. Jimtaisong , “Photoprotection of Natural Flavonoids,” Journal of Applied Pharmaceutical Science 9 (2013): 129–141.

[67] G. F. dos Santos , R. O. Defendi , and E. Düsman , “Valorization of Wastes From the Juice Passion Fruit Production Industry: Extraction of Bioactive Compounds From Seeds, Antioxidant, Photoprotective, and Antiproliferative Activities,” Waste and Biomass Valorization 14 (2022): 1233–1250.

[68] D. Pauluk , A. K. Padilha , N. M. Khalil , and R. M. Mainardes , “Chitosan‐coated Zein Nanoparticles for Oral Delivery of Resveratrol: Formation, Characterization, Stability, Mucoadhesive Properties and Antioxidant Activity,” Food Hydrocolloids 94 (2019): 411–417.

[69] S. Chen , Y. Han , L. Jian , W. Liao , Y. Zhang , and Y. Gao , “Fabrication, Characterization, Physicochemical Stability of Zein‐chitosan Nanocomplex for Co‐encapsulating Curcumin and Resveratrol,” Carbohydrate Polymers 236 (2020): 1–9. 10.1016/j.carbpol.2020.116090 32172893

[70] H. Yang , Y. Mu , D. Zheng , et al., “Caseinate‐coated Zein Nanoparticles as Potential Delivery Vehicles for Guavinoside B From Guava: Molecular Interactions and Encapsulation Properties,” Food Chemistry 456 (2024): 140066. 10.1016/j.foodchem.2024.140066 38901076

[71] Q. Liu , Y. Jing , C. Han , H. Zhang , and Y. Tian , “Encapsulation of Curcumin in Zein/Caseinate/Sodium Alginate Nanoparticles With Improved Physicochemical and Controlled Release Properties,” Food Hydrocolloids 93 (2019): 432–442.

[72] R. Nunes , A. Baião , D. Monteiro , J. das Neves , and B. Sarmento , “Zein Nanoparticles as Low‐cost, Safe, and Effective Carriers to Improve the Oral Bioavailability of Resveratrol,” Drug Delivery and Translational Research 10 (2020): 826–837. 10.1007/s13346-020-00738-z 32207071

[73] L. A. A. Campos , A. F. Silva Neto , M. C. S. Noronha , M. F. de Lima , I. M. F. Cavalcanti , and N. S. Santos‐Magalhães , “Zein Nanoparticles for Drug Delivery: Preparation Methods and Biological Applications,” International Journal of Pharmaceutics 635 (2023): 122754. 10.1016/j.ijpharm.2023.122754 36812950

[74] Y. Zhang , Y. Niu , Y. Luo , et al., “Fabrication, Characterization, and Antimicrobial Activities of Thymol‐loaded Zein Nanoparticles Stabilized by Sodium Caseinate–chitosan Hydrochloride Double Layers,” Food Chemistry 142 (2014): 269–275. 10.1016/j.foodchem.2013.07.058 24001841

[75] D. K. Takma , S. Bozkurt , M. Koç , F. Korel , and H. Nadeem , “Characterization and Encapsulation Efficiency of Zein Nanoparticles Loaded With Chestnut Fruit Shell, Cedar and Sweetgum Bark Extracts,” Food Hydrocolloids Health 4 (2023): 100151.

[76] S. Zhang and Y. Han , “Preparation, Characterisation, and Antioxidant Activities of Rutin‐loaded Zein‐caseinate Nanoparticles,” PLoS One 13 (2018): e0194951. 10.1371/journal.pone.0194951 PMC5868844 29579133

[77] S. Goenka and S. R. Simon , “Depigmenting Effect of Xanthohumol From Hop Extract in MNT‐1 Human Melanoma Cells and Normal Human Melanocytes,” Biochemical and Biophysical Reports 26 (2021): 100955. 10.1016/j.bbrep.2021.100955 PMC7910408 33681480

[78] J. F. Stevens and J. E. Page , “Xanthohumol and Related Prenylflavonoids From Hops and Beer: To Your Good Health!,” Phytochemistry 65 (2004): 1317–1330. 10.1016/j.phytochem.2004.04.025 15231405

[79] J. F. Stevens , C. L. Miranda , D. R. Buhler , and M. L. Deizer , “Chemistry and Biology of Hop Flavonoids,” Journal of the American Society of Brewing Chemists 56 (1998): 136–145.

[80] C. L. Miranda , J. F. Stevens , A. Helmrich , et al., “Antiproliferative and Cytotoxic Effects of Prenylated Flavonoids From Hops (*Humulus lupulus*) in Human Cancer Cell Lines,” Food and Chemical Toxicology 37 (1999): 271–285. 10.1016/s0278-6915(99)00019-8 10418944

[81] I. E. Logan , C. L. Miranda , M. B. Lowry , C. S. Maier , J. F. Stevens , and A. F. Gombart , “Antiproliferative and Cytotoxic Activity of Xanthohumol and Its Non‐Estrogenic Derivatives in Colon and Hepatocellular Carcinoma Cell Lines,” International Journal of Molecular Sciences 20 (2019): 1203. 10.3390/ijms20051203 PMC6429097 30857300

[82] N. Ghobadi‐Oghaz , A. Asoodeh , and M. Mohammadi , “Fabrication, Characterization and *In Vitro* Cell Exposure Study of Zein‐chitosan Nanoparticles for co‐delivery of Curcumin and Berberine,” International Journal of Biological Macromolecules 204 (2022): 576–586. 10.1016/j.ijbiomac.2022.02.041 35157902

[83] W. Brand‐Williams , M. E. Cuvelier , and C. Berset , “Use of a Free Radical Method to Evaluate Antioxidant Activity,” LWT—Food Science and Technology 28 (1995): 25–30.

[84] P. A. Bressiani , G. R. F. de Lima , E. Düsman , and L. T. D. Tonin , “Cytotoxic and Antioxidant Activities of *Tamarindus Indica* Pulp Extract From Brazil,” Journal of Food Measurement and Characterization 15 (2021): 2743–2749.

[85] M. S. M. Rufino , R. E. Alves , E. S. Brito , et al., “Metodologia Científica: Determinação da atividade antioxidante total em frutas pela captura do radical livre ABTS+,” Embrapa—Comunicado Técnico on line 128 (2007): 1–4.

[86] L. L. Stookey , “Ferrozine—A New Spectrophotometric Reagent for Iron,” Analytical Chemistry 42 (1970): 779–781.

[87] M. Borosky , J. V. Visentainer , S. M. Cottica , and D. R. de Morais , “Antioxidantes: Princípios E Métodos Analíticos,” (Appris, Curitiba 2015).

[88] V. L. Singleton and J. A. Rossi , “Colorimetry of Total Phenolics With Phosphomolybdic‐phosphotungstic Acid Reagents,” American Journal of Enology and Viticulture 16 (1965): 144–158.

[89] R. G. Woisky and A. Salatino , “Analysis of Propolis: Some Parameters and Procedures for Chemical Quality Control,” Journal of Apicultural Research 37 (1998): 99–105.

[90] L. J. Wang , Y. Q. Hu , S. W. Yin , X. Q. Yang , F. R. Lai , and S. Q. Wang , “Fabrication and Characterization of Antioxidant Pickering Emulsions Stabilized by Zein/Chitosan Complex Particles (ZCPs),” Journal of Agricultural and Food Chemistry 63 (2015): 2514–2524. 10.1021/jf505227a 25636210

[91] J. J. Ma , Y. G. Yu , S. W. Yin , C. H. Tang , and X. Q. Yang , “Cellular Uptake and Intracellular Antioxidant Activity of Zein/Chitosan Nanoparticles Incorporated With Quercetin,” Journal of Agricultural and Food Chemistry 66 (2018): 12783–12793. 10.1021/acs.jafc.8b04571 30406660

[92] H. Li , Y. Xu , X. Suna , et al., “Stability, Bioactivity, and Bioaccessibility of Fucoxanthin in Zein‐caseinate Composite Nanoparticles Fabricated at Neutral pH by Antisolvent Precipitation,” Food Hydrocolloids 84 (2018): 379–388.

[93] T. Mosmann , “Rapid Colorimetric Assay for Cellular Growth and Survival: Application to Proliferation and Cytotoxicity Assays,” Journal of Immunological Methods 65 (1983): 55–63. 10.1016/0022-1759(83)90303-4 6606682

